# Supplementary material for: Caenorhabditis elegans sperm carry a histone-based epigenetic memory of both spermatogenesis and oogenesis
Source: Nat Commun. 2018 Oct 17;9:4310. doi: 10.1038/s41467-018-06236-8 (PMC6193031; doi:10.1038/s41467-018-06236-8)
Supplement: Supplementary file 1 — Supplementary Information [file 41467_2018_6236_MOESM1_ESM.pdf]

Caenorhabditis elegans sperm carry a histone-based epigenetic memory of both spermatogenesis and oogenesis

Tabuchi et al.

Supplementary Figure 1

Supplementary Figure 2

Supplementary Figure 3

Supplementary Figure 4

Supplementary Figure 5

Supplementary Figure 6

Supplementary References

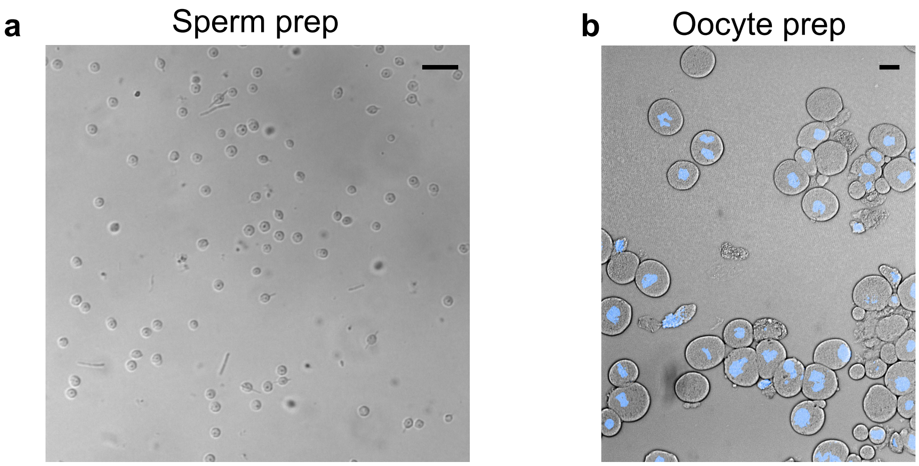

Supplementary Figure 1 | Preparations of sperm and oocytes. (**a**) Representative image of purified sperm. (**b**) Representative image of isolated oocytes with DNA stained with DAPI (blue). Estimation of purity is described in Methods. Scale bars represent 20 µm.

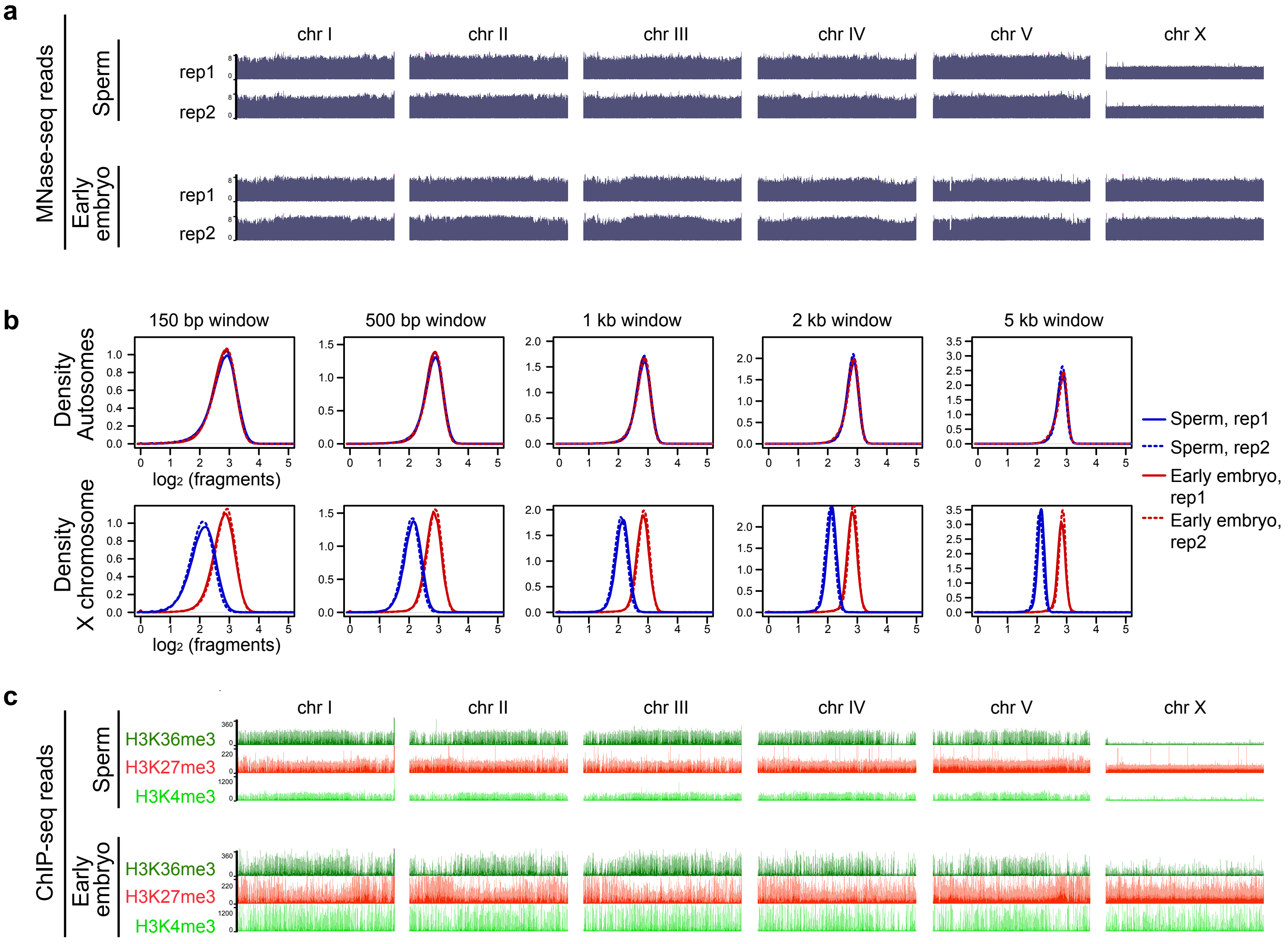

Supplementary Figure 2 | MNase-seq and ChIP-seq analyses to evaluate the presence of nucleosomes across the sperm genome. (a) MNase-seq across all 6 chromosomes, showing genome-wide occupancy of nucleosomes in both sperm and early embryos. Regions occupied by nucleosomes yield MNase-protected fragments of ~150 bp DNA, while regions devoid of nucleosomes (e.g. packaged instead with protamines) lack MNase-protected fragments. Coverage by MNase-seq fragments corresponding to mononucleosomes is displayed in the UCSC genome browser after fragments of <140 bp were computationally filtered out. The y-axis shows normalized fragment coverage. Sperm show reduced signal on the X chromosome compared to the autosomes, as expected, since 50% of sperm from XO males do not have an X chromosome. **(b)** Density plots showing distributions of $\log_2$ (fragment coverage) for autosomes and the X chromosome from sperm and early embryos binned to various window sizes (150 bp, 500 bp, 1 kb, 2 kb, and 5 kb). The shapes of density plots show if two datasets are similarly distributed or not; the area under each curve adds up to 1. We found that the distribution of fragment coverage on autosomes is identical for sperm and early embryos, suggesting that nucleosome occupancy on the autosomes is similar between sperm and early embryos. X-chromosome coverage from sperm is ~2-fold lower compared to early embryos, as expected. Two biological replicates each for sperm and early embryo MNase-seq were obtained. **(c)** The genome-wide distribution of H3K36me3, H3K27me3, and H3K4me3 ChIP-seq. The y-axes show normalized ChIP-seq read counts.

Supplementary Figure 3

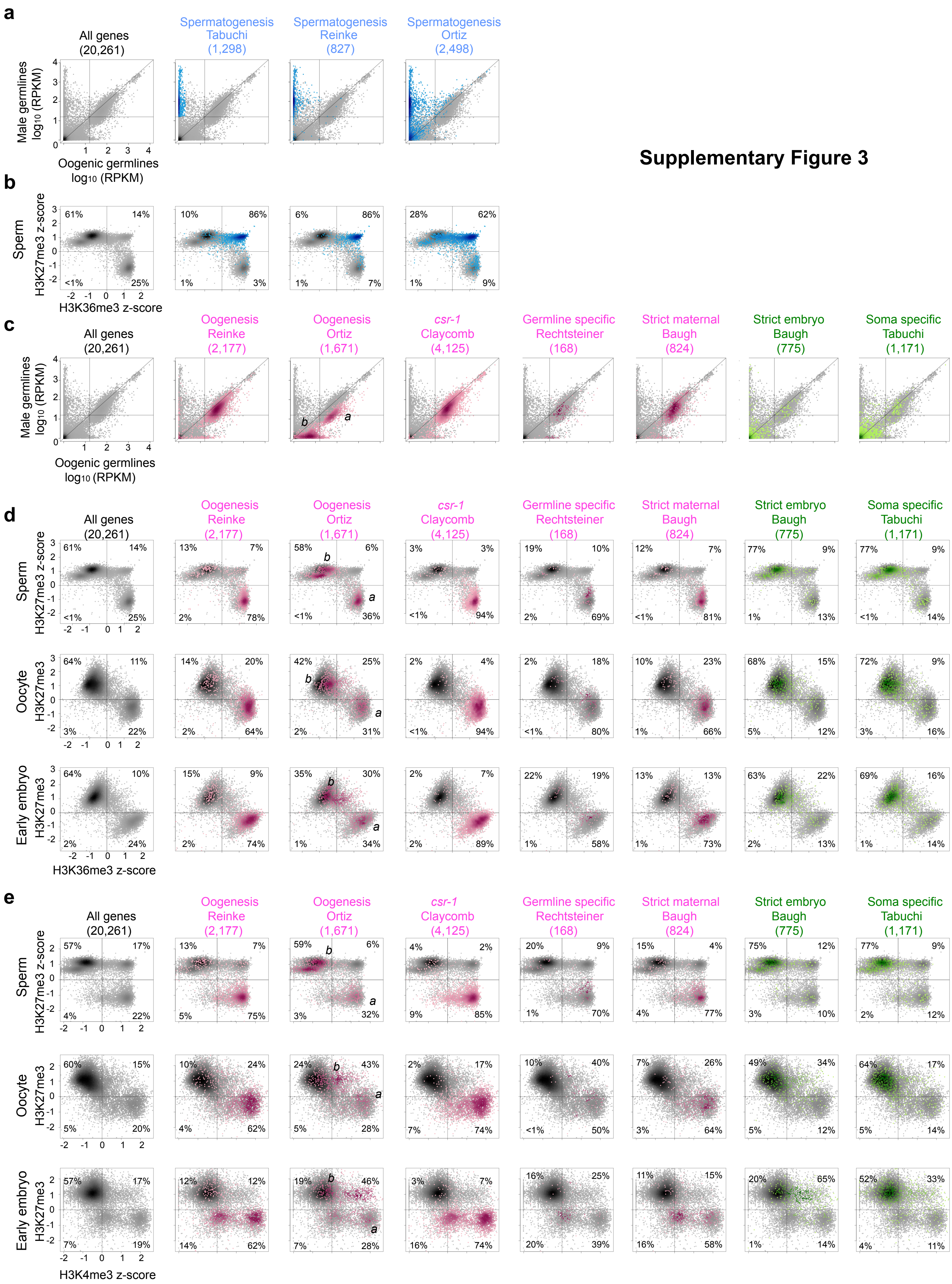

Supplementary Figure 3 | Comprehensive analyses of RNA-seq and ChIP-seq data, highlighting gene sets defined in various publications. (**a**,**b**) RNA-seq analysis of spermatogenic vs. oogenic germlines (**a**) and ChIP-seq analysis of H3K36me3 vs. H3K27me3 in sperm (**b**), highlighting in blue spermatogenesis-specific gene sets defined in Methods (Tabuchi), and spermatogenesis-enriched gene sets defined by Reinke et al. (2004)[1] and by Ortiz et al. (2014)[2], noting the % of genes in each quadrant. RNA-seq axes show log-transformed RPKMs after adding a pseudo-count of 1. The grey lines show the cut-off for expressed genes (RPKM of 15, Methods). (**c**) RNA-seq data, highlighting gene sets related to oogenesis[1–5] (pink) and somatic cells[5] (green), as discussed in Methods. The majority of genes in pink appear just below the diagonal line, demonstrating that 'oogenesis genes' are transcribed in both oogenic and spermatogenic germlines and that transcript levels are higher in oogenic germlines. We refer to these as 'oogenesis-enriched genes' or 'oogenesis genes' for short. We were not able to identify 'oogenesis-specific' genes with the same confidence cut-off as was used for spermatogenesis-specific genes (Methods). We note that the oogenesis gene set defined by Ortiz is composed of two sub-groups ('*a*' and '*b*'). Genes in the '*a*' subgroup are marked with the active modifications H3K36me3 and H3K4me3, as expected for oogenesis-enriched genes. Genes in the '*b*' subgroup are marked with the repressive modification H3K27me3 in both oocytes and early embryos, similar to genes expressed in somatic cells. Thus, we speculate that Ortiz oogenesis gene subgroup '*b*' may result from somatic cell contamination. Axes show log-transformed RPKMs after adding a pseudo-count of 1. The grey lines show the cut-off for expressed genes (RPKM of 15, Methods). (**d**,**e**) ChIP-seq scatter plots showing gene-body H3K36me3 (**d**) or promoter H3K4me3 (**e**) vs. gene-body H3K27me3 from sperm, oocytes, and early embryos, showing the same gene sets as analyzed in panel **c**. We conclude that differently defined sets of 'oogenesis genes' (pink) from various publications based on different types of data are expressed in both oogenic and spermatogenic germlines, and are marked with active modifications (H3K36me3 and H3K4me3) in both oocytes and sperm.

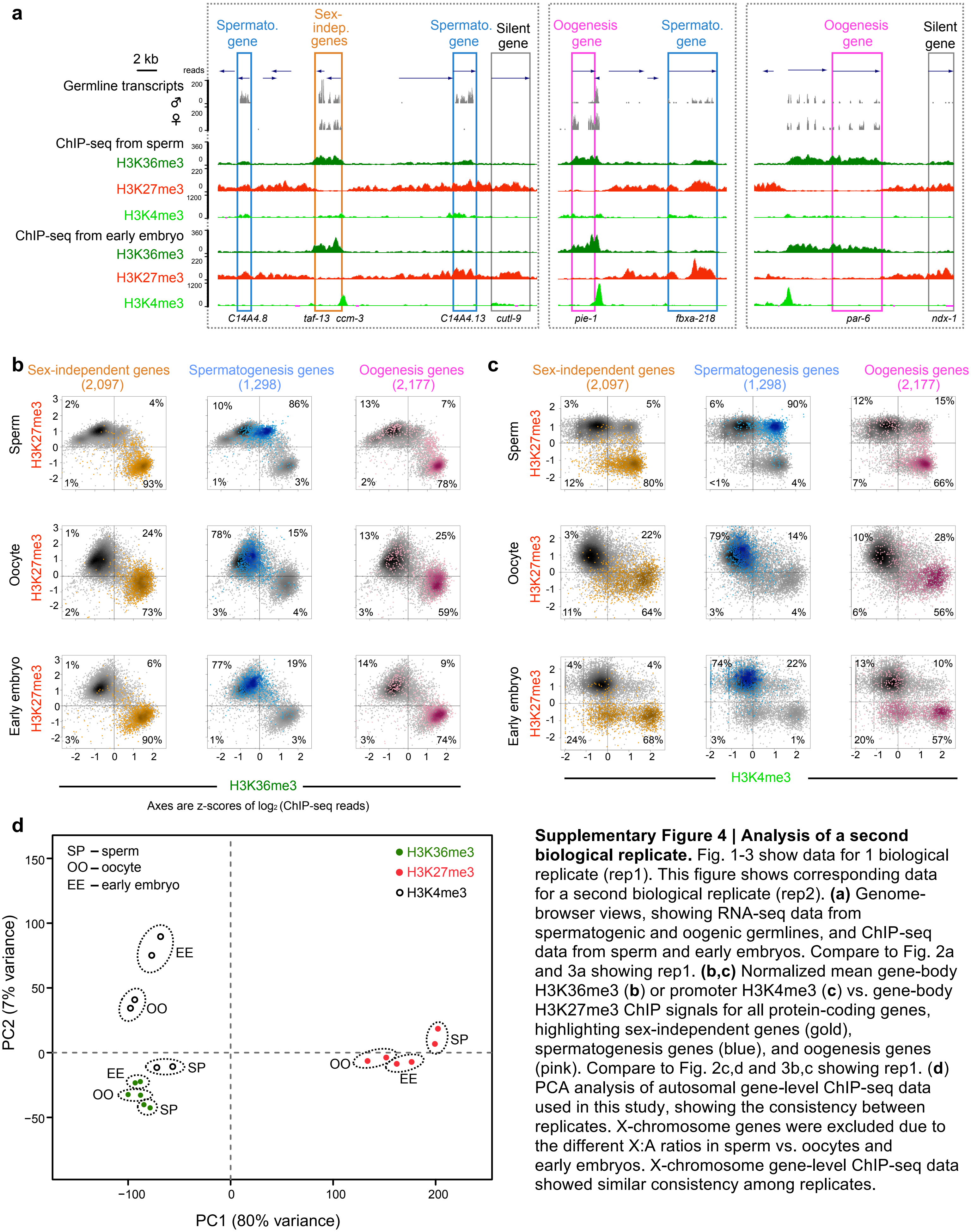

Supplementary Figure 4 | Analysis of a second biological replicate. Fig. 1-3 show data for 1 biological replicate (rep1). This figure shows corresponding data for a second biological replicate (rep2). **(a)** Genome-browser views, showing RNA-seq data from spermatogenic and oogenic germlines, and ChIP-seq data from sperm and early embryos. Compare to Fig. 2a and 3a showing rep1. **(b,c)** Normalized mean gene-body H3K36me3 (**b**) or promoter H3K4me3 (**c**) vs. gene-body H3K27me3 ChIP signals for all protein-coding genes, highlighting sex-independent genes (gold), spermatogenesis genes (blue), and oogenesis genes (pink). Compare to Fig. 2c,d and 3b,c showing rep1. (**d**) PCA analysis of autosomal gene-level ChIP-seq data used in this study, showing the consistency between replicates. X-chromosome genes were excluded due to the different X:A ratios in sperm vs. oocytes and early embryos. X-chromosome gene-level ChIP-seq data showed similar consistency among replicates.

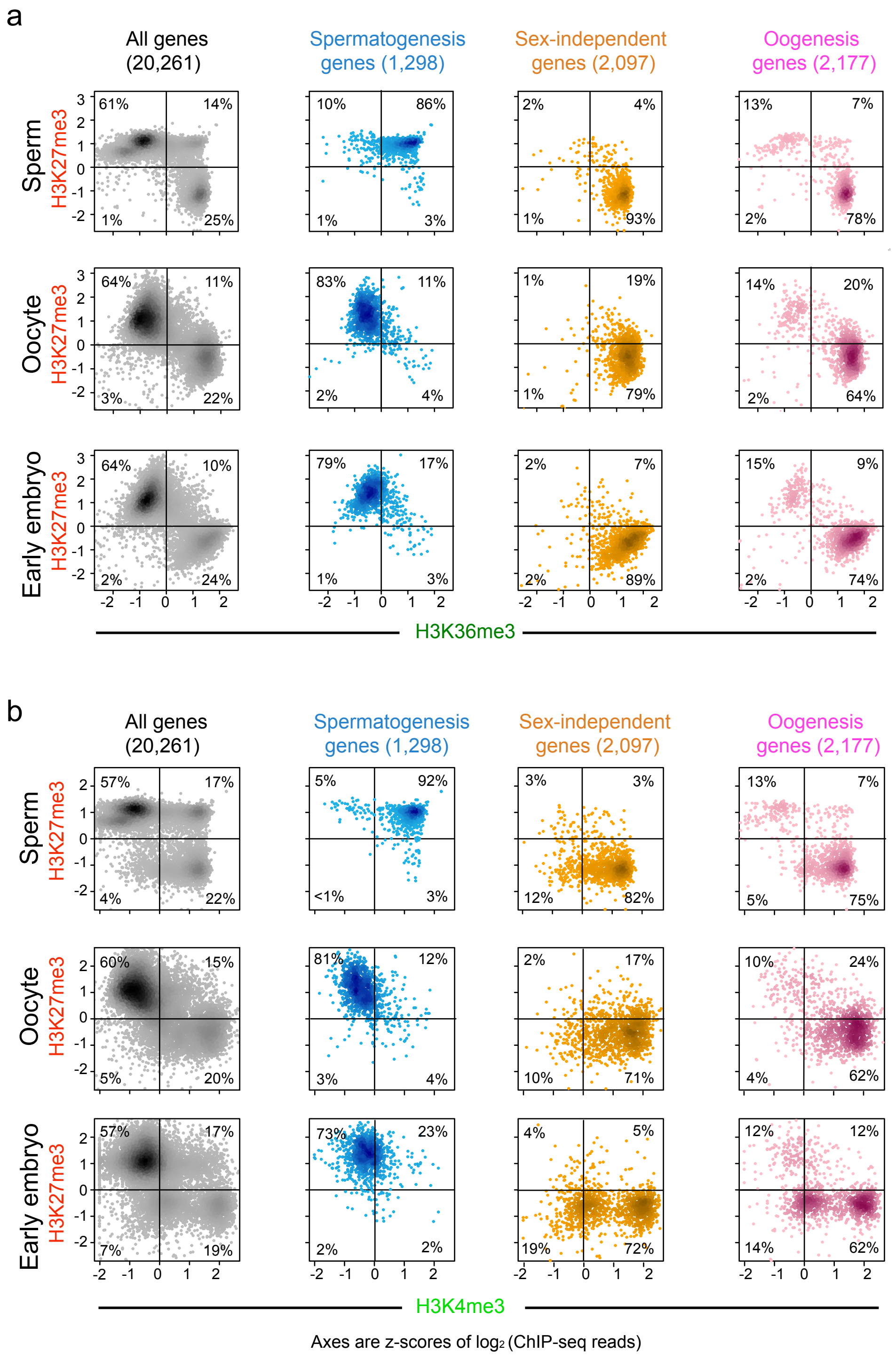

Supplementary Figure 5 | Scatter plots from Fig. 2 and 3, highlighting each class of genes in separate panels. The normalized gene-body H3K36me3 (**a**) or promoter H3K4me3 (**b**) vs. gene-body H3K27me3 ChIP signals for all protein-coding genes (grey), spermatogenesis genes (blue), sex-independent genes (gold), and oogenesis genes (pink).

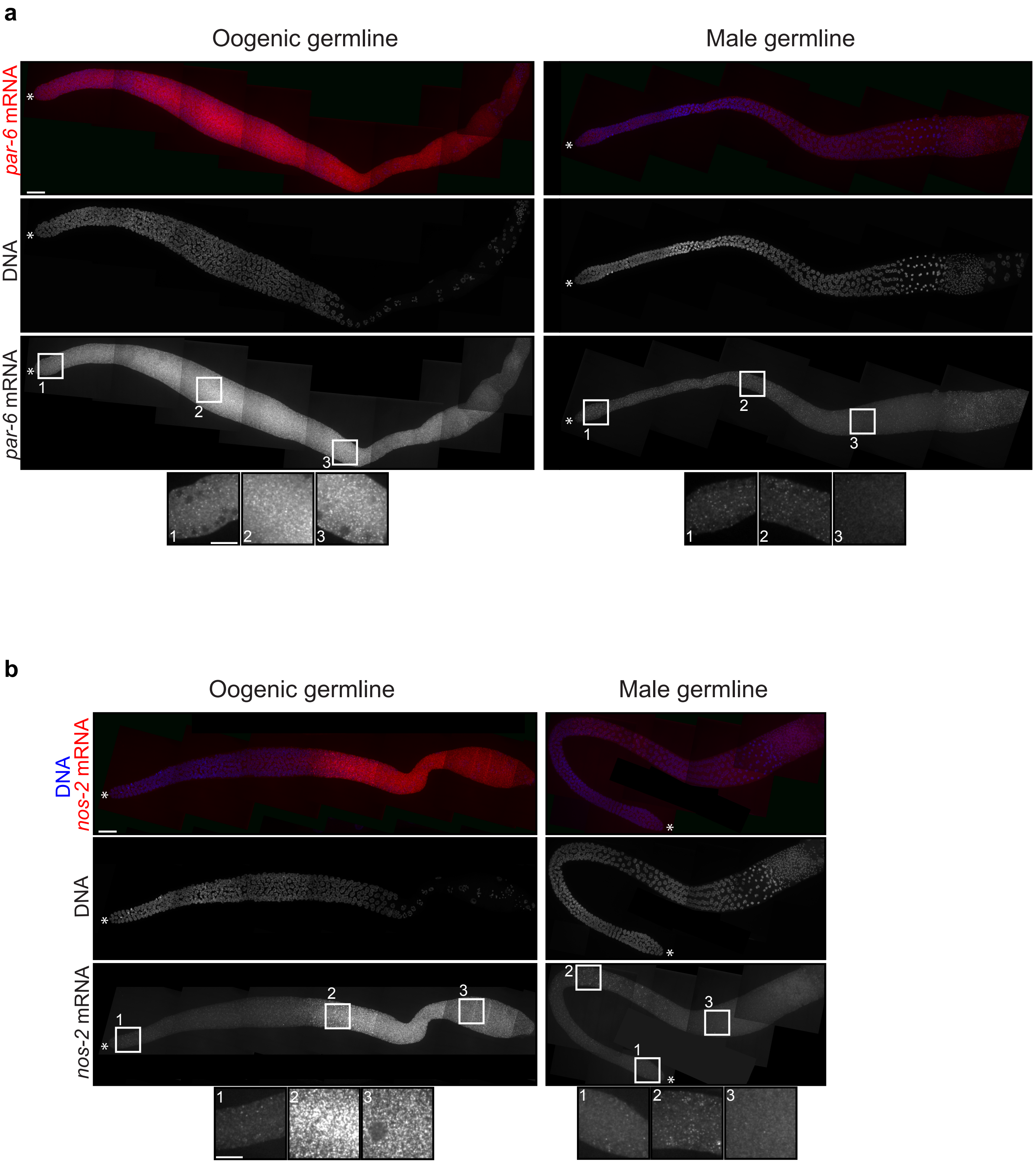

Supplementary Figure 6 | Oogenic transcripts are detected in male germlines by smFISH. smFISH analysis in oogenic and male germlines of RNA from well-known genes transcribed during oogenesis to guide early embryo development: *par-6* **(a)** and *nos-2* **(b)**. Panels 1-3 at the bottom of each set of images show high-magnification images (projections of 3 slices) of the boxed regions in the lower-magnification images (z-stack projection). 10-60 gonads were visually examined under the microscope, and at least 3 germlines were imaged. The distal tip is marked with an *. Scale bars represent 20 μm for the top panels and 10 μm for the bottom panels.

Supplementary References

1. Reinke, V., Gil, I. S., Ward, S. & Kazmer, K. Genome-wide germline-enriched and sex-biased expression profiles in *Caenorhabditis elegans*. *Development* **131,** 311–323 (2004).
2. Ortiz, M. A., Noble, D., Sorokin, E. P. & Kimble, J. A new dataset of spermatogenic vs. oogenic transcriptomes in the nematode *Caenorhabditis elegans*. *G3* **4,** 1765–1772 (2014).
3. Claycomb, J. M. *et al.* The argonaute CSR-1 and its 22G-RNA cofactors are required for holocentric chromosome segregation. *Cell* **139,** 123–134 (2009).
4. Rechtsteiner, A. *et al.* The histone H3K36 methyltransferase MES-4 acts epigenetically to transmit the memory of germline gene expression to progeny. *PLoS Genet* **6,** e1001091 (2010).
5. Baugh, L. R., Hill, A. A., Slonim, D. K., Brown, E. L. & Hunter, C. P. Composition and dynamics of the *Caenorhabditis elegans* early embryonic transcriptome. *Development* **130,** 889–900 (2003).
