# Supplementary material for: Caenorhabditis elegans sperm carry a histone-based epigenetic memory of both spermatogenesis and oogenesis
Source: Nat Commun. 2018 Oct 17;9:4310. doi: 10.1038/s41467-018-06236-8 (PMC6193031; doi:10.1038/s41467-018-06236-8)
Supplement: Supplementary file 3 — Description of Additional Supplementary Files [file 41467_2018_6236_MOESM3_ESM.pdf]

Description of Additional Supplementary Files

File Name: Supplementary Data 1
Description: Gene sets used in this study. Sex-independent (2,097 genes defined in Methods), Spermatogenesis-specific (1,298 genes defined in Methods), Spermatogenesis-enriched (827 genes, Reinke et al. Development, 2004), Sperm-enriched (2,498 genes, Ortiz et al. G3, 2014), Oogenesis-enriched (2,177 genes, Reinke et al. Development, 2004), Oogenesis-enriched (1,671 genes, Ortiz et al. G3, 2014), CSR-1-bound (4,125 genes targeted by small RNAs bound to the Argonaute protein CSR-1, Claycomb et al. Cell, 2009), Germline-specific (168 genes, Rechtsteiner et al. PLoS Genetics, 2010), Strict-maternal (824 genes detected at the earliest time point in early embryos, Baugh et al. Development, 2003), Soma-specific (1,171 genes defined in Methods), Strict-embryo (775 genes that increase in expression at some point during embryogenesis, Baugh et al. Development, 2003), Silent (410 serpentine receptor genes that are expressed in a few mature neurons and are not expected to be expressed in germlines, Kolasinska-Zwierz et al. Nature Genetics, 2009). Genes included in each set are indicated with a “1”.
